# Effects of Tetraethyl Orthosilicate (TEOS) on the Light and Temperature Stability of a Pigment from *Beta vulgaris* and Its Potential Food Industry Applications

**DOI:** 10.3390/molecules191117985

**Published:** 2014-11-05

**Authors:** Gustavo A. Molina, Angel Ramon Hernández-Martínez, Manuel Cortez-Valadez, Fernando García-Hernández, Miriam Estevez

**Affiliations:** 1 Licenciatura en Tecnología, Centro de Física Aplicada y Tecnología Avanzada (CFATA), Universidad Nacional Autónoma de México, Campus Juriquilla, Querétaro, Querétaro C.P. 76230, Mexico; E-Mail: gustavomolina21@gmail.com; 2 Centro de Investigación y de Estudios Avanzados del IPN, Unidad Querétaro, Apdo. Postal 1-798, Querétaro, Querétaro C.P. 76001, Mexico; E-Mail: angel.ramon.hernandez@gmail.com; 3 Centro de Investigación en Física, Universidad de Sonora, Apdo. Postal 5-88, Hermosillo, Sonora C.P. 83190, Mexico; E-Mail: manuelcortez@live.com; 4 Eisiaba, Instituto Tecnológico de Estudios Superiores Monterrey, Campus Querétaro, Querétaro C.P. 76130, Mexico; E-Mail: fergar17@prodigy.net.mx; 5 Centro de Física Aplicada y Tecnología Avanzada (CFATA), Universidad Nacional Autónoma de México, Campus Juriquilla, Querétaro, Querétaro C.P. 76130, Mexico

**Keywords:** food-pigment, *Beta vulgaris*, pigment, tetraethyl orthosilicate, red beet

## Abstract

A novel, simple and inexpensive modification method using TEOS to increase the UV light, pH and temperature stability of a red-beet-pigment extracted from *Beta vulgaris* has been proposed. The effects on the molecular structure of betalains were studied by FTIR spectroscopy. The presence of betacyanin was verified by UV-Vis spectroscopy and its degradation in modified red-beet-pigment was evaluated and compared to the unmodified red-beet-pigment; performance improvements of 88.33%, 16.84% and 20.90% for UV light, pH and temperature stability were obtained, respectively,. Measurements of reducing sugars, phenol, and antioxidant contents were performed on unmodified and modified red-beet-pigment and losses of close to 21%, 54% and 36%, respectively, were found to be caused by the addition of TEOS. Polar diagrams of color by unmodified and modified red-beet-pigment in models of a beverage and of a yogurt were obtained and the color is preserved, although here is a small loss in the chromaticity parameter of the modified red-beet-pigment.

## 1. Introduction

Natural colorants are Generally Regarded as Safe (GRAS) substances. Therefore, they are more desirable than the synthetic ones for industrial or commercial applications as food additives. However, they are more expensive to obtain and usually they have lower stability, restricting sometimes their practical use as colorants [1,2]. Therefore, natural pigment applications are typically limited to areas where particular reliability is required, such as food and cosmetics, where color is one of the most important attributes for product acceptance [3,4,5,6,7,8].

Currently, the interest in using natural colorants has increased because of their non-toxicity and beneficial health effects, mainly as antioxidants [9,10,11,12]. The number of synthetic dyes currently allowed by the FDA has been reduced from 700 to 7 [13,14,15]. This is due to their negative effects on the environment and their links to allergic, toxic, carcinogenic, and harmful responses.

Betalains have no toxic effects on the human body and are seen as a natural and safe alternative to synthetic red coloring [16,17,18]. The betalains are a group of water-soluble nitrogen-containing pigments that are yellow, orange, pink, red and purple colored [19,20], with a tendency to easily degrade in solution. They also present activity as antioxidants and scavengers of radicals which contribute to the onset of diverse human diseases [21,22,23]. Attoe and Von Elbe reported that their stability is affected during processing and storage [24].

In the food industry, there is a growing tendency to replace synthetic dyes because of consumer preference [14]. Betalains are barely used, although these water-soluble pigments are stable from 3 to 7 pH and could be used as a low acid food coloring [19]. The name betalains describes two main groups, the betacyanins (λ = 540 nm) and betaxanthins (λ = 480 nm). The disadvantages of natural colorants are their instability to light, temperature, pH, oxygen and water [25,26,27,28]. If natural colorants’ stability were improved, they could be widely used in many applications.

Incorporation of organic pigments into inorganic hosts sometimes enhances their stability. For example, cationic pigments have been stabilized by inserting them among the layers of cation exchangeable clays or zeolites. It has also been reported that the stability of natural anthocyanin pigments is generally enhanced by complexation with the cation exchangeable clay montmorillonite [29].

We proposed to use a sol-gel precursor such as tetraethyl orthosilicate (TEOS) on a natural colorant to improve its stability. TEOS—as an alkoxide—was used because it reacts under acidic conditions with the carbonyl groups of the betalain molecule to form new carbonyl-oxygen-silicon bonds, giving more stability to this natural colorant. This novel modified pigment can be used in the food industry, cosmetics and paints in general. We approach the stability problems by investigating the effect of TEOS in a red-beet-pigment obtained from *Beta vulgaris*. The work includes a study of its thermal, photo, and pH stabilities, as well as color; and reducing sugars, phenol, and antioxidant contents. We also observed the modification in the carbonyl group using FTIR spectroscopy and confirmed it by software simulation of the modified betalain pigment molecule.

## 2. Results and Discussion

### 2.1. UV-Light Test

**Figure 1:**
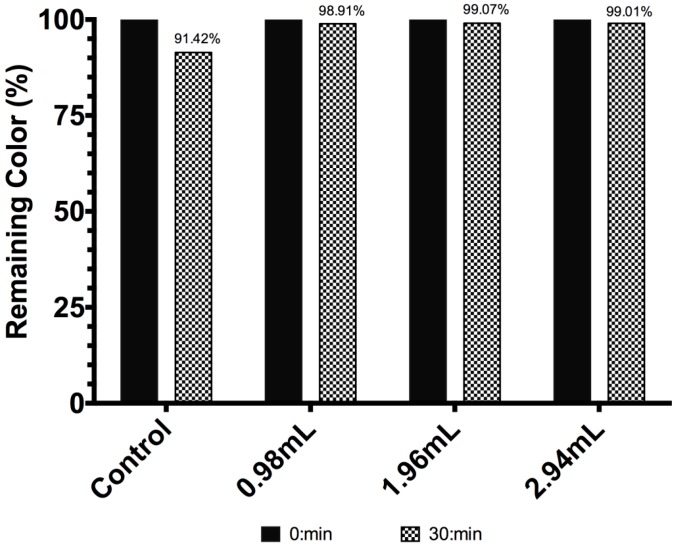
Betacyanin concentration (remaining color percent) for *Beta vulgaris* (Control) and modified *Beta vulgaris*, exposed to UV-light radiation.

The UV-light stability of betacyanin and modified betacyanin is shown in Figure 1. The color concentration of unmodified betalain showed a decrease of 8.57% after thirty minutes of UV-light irradiation. On the other hand, for betalain modified with 0.98, 1.96 and 2.94 mL of TEOS the decreases in the color concentration were 1.09%, 0.92% and 0.99%, respectively, corresponding to degradation percentages of 12.72%, 10.74%, and 11.55%, taking the original degradation of unmodified betalain molecules as 100%. These values represent a performance improvement of 87.28%, 89.26% and 88.45% for BE_1_, BE_2_ and BE_3_, respectively. This dramatic improvement can be explained if the original betalain molecule is conceived as a compound surrounded by SiO_2_ molecules, which have the particularity to scatter light to reduce the probability of a photon to excite the π electrons of the chromophore pigment to break its bonds. The differences in the remaining color between BE_2_ and BE_3_ are within the margin of error, so it is not possible to establish a tendency based on the volume of TEOS used.

### 2.2. PH Behavior Test

**Figure 2:**
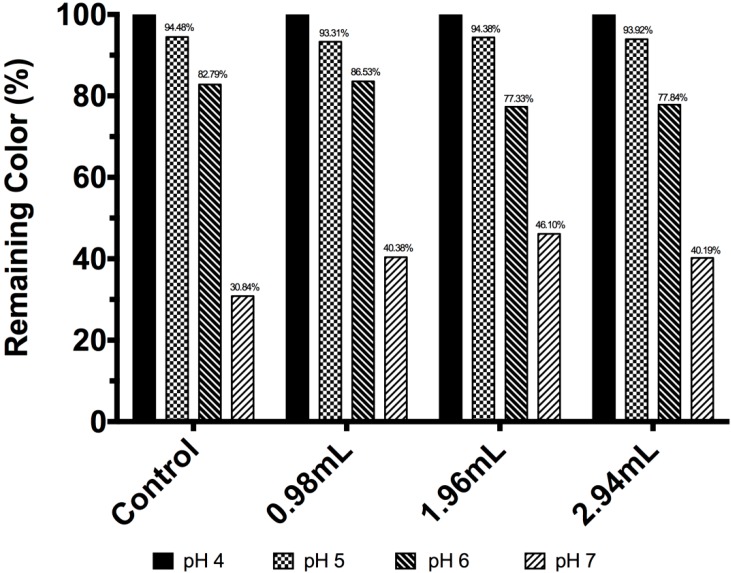
Betacyanin concentration expressed in remaining color percentage for *Beta vulgaris* and modified *Beta vulgaris* at different pH values.

The test started at pH 4. As seen in Figure 2, the unmodified betalain color concentration showed a decline of 5.52%, 17.21% and 69.16% at pH 5, 6 and 7, respectively. A similar case was observed for BE_1_, which showed a decline of 6.69%, 13.47% and 59.62% at pH 5, 6 and 7, respectively. For BE_2_ and BE_3_, similar tendencies were observed; these color concentration declines could be explained as a result of hydrolysis of the betalain molecule to betalamic acid and cyclodopa-5-*O*-glycoside [30].

### 2.3. Temperature Test

**Figure 3:**
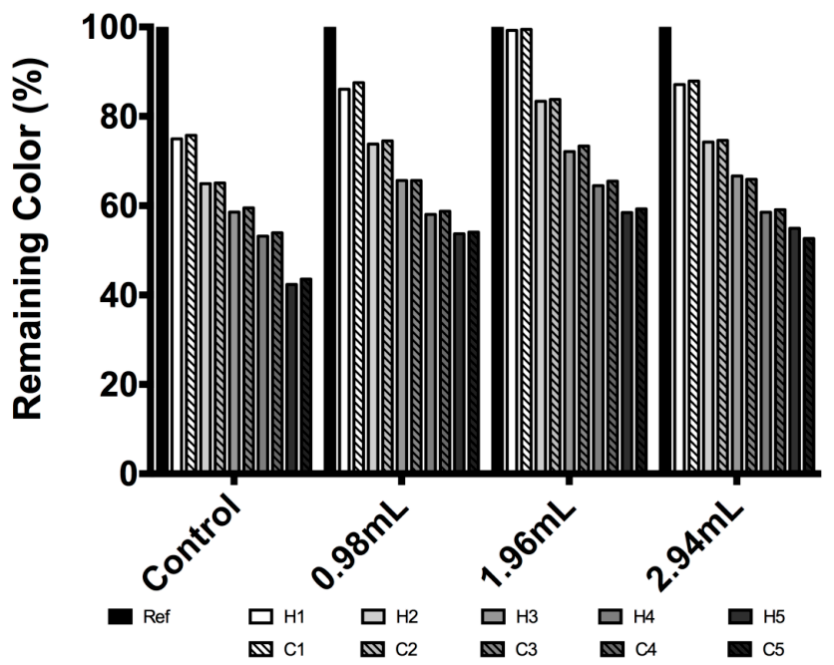
Betacyanin concentration expressed in remaining color percentage for temperature stability evaluation for *Beta vulgaris* and modified *Beta vulgaris*.

Figure 3 shows a temperature stability test. In the first heat-cold cycle of the unmodified pigment, a decay of 25% for a heat half-cycle and an upswing of 0.8% by a cold half-cycle were observed. Hence a percentage of decrease was observed for each of the five heat-cold cycles. The decreases, for the first to the fifth cycles, were 24.2%, 34.9%, 40.7%, 46.06%, and 56.62%, respectively.

An interesting fact worth highlighting is that the 1% increase in the concentration found in each cold half-cycle of unmodified betalain may be explained by a partially reversible reaction that involves a Schiff base condensation of the amine of cyclodopa-5-*O*-glycoside with the aldehyde of betalamic acid; it is well known that temperature and pH affect the reversibility of Schiff base reactions [24,29,30,31,32,33].

**Figure 4:**
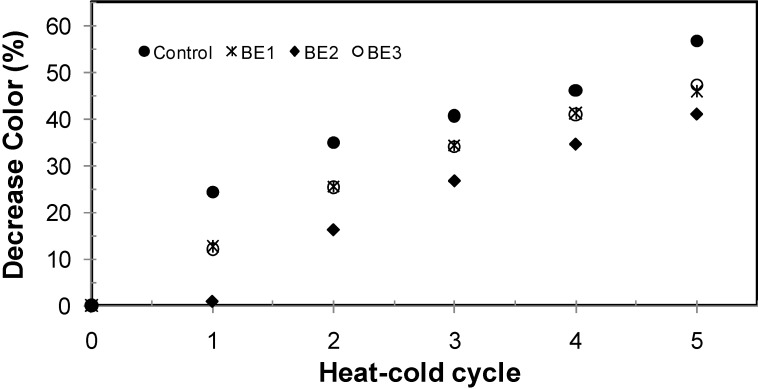
Decrease of color percentage as a function of heat-cold cycles; ● unmodified betalain; * BE_1_; ♦ BE_2_; ○ BE_3_.

For modified betalain this increase in the cold half-cycle was not observed in any case, instead a 1st-derivative—with respect to cycles—was found, as shown in Figure 4; the total decay at the end of five heat-cold cycles for betalain color concentration were 56.62%, 45.94%, 40.94% and 47.35% for unmodified betalain, BE_1_, BE_2_ and BE_3_, respectively; these could be a consequence of heat dissipation generated by the silicate particles.

### 2.4. Reducing Sugars, Phenol, and Antioxidant Content Measurements.

Table 1 shows total reducing sugars, total phenol content and antioxidant content of unmodified and modified-pigment. As seen, the largest loss in the modified pigment occurred in the total phenols, although 47% of the total phenols were retained, which is a valuable result considering that some polyphenols have antimicrobial activity [34], in addition to having antimutagenic activity [35], and have proven their ability to act as hydrogen donors or as metal ion chelators, such as iron or copper oxidation inhibiting low density lipoprotein—which are involved in the pathogenesis of coronary heart disease [36].

**Table 1:** Nutritional Value Contents for Unmodified and Modified Red-Beet-Pigment

Sample	Total Antioxidant ^a^ (mg/g)	Total Phenols ^b^ (mg/g)	Total Reducing sugars ^c^ (mg/g)
Unmodified red-beet-pigment	33.30 ± 0.3	35.3 ± 4.05	26.77 ± 0.9
Modified red-beet-pigment	21.25 ± 0.2	16.3 ± 0.44	21.13 ± 4.8
Nutritional reduction	12.05	19	5.64
%Nutritional reduction	36.2	53.8	21.1

**^a^** mg/L of ascorbic acid equivalent; **^b^** mg/g of gallic acid equivalent; **^c^** mg/L of sugar.

The antioxidants present in beverages may protect sensory quality [37] and reduce the negative effects of cellular oxidative stress on the final consumer. Therefore it is relevant that the percentage of conservation is of 63.8%, representing only a 36.2% loss.

### 2.5. Model Food Applications

**Figure 5:**
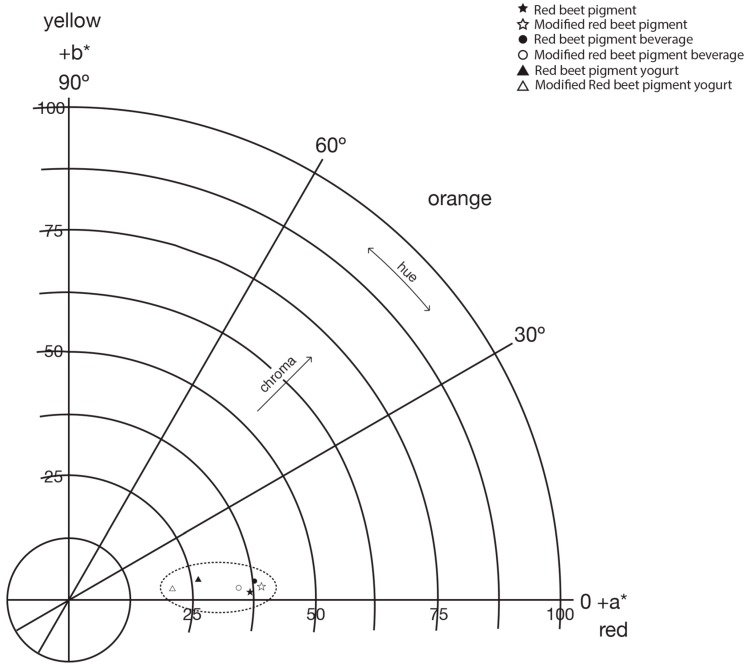
Polar color diagram for red beet pigments before and after the chemical modification process and applied to beverage and yogurt model systems.

Figure 5 shows the polar color diagram of unmodified and modified betalain in a beverage and a yogurt. The difference between colors can be described by the total distance between those colors in the three dimensional CIElab color space known as ∆E. A color difference in the range from 0–1.5 is considered small—the colors can be considered almost identical—a difference from 1.5–5 can be distinguished and for a difference higher than 5 is visually evident [38]. The difference between unmodified and modified red-beet-pigments is ∆E = 2.63; hence adding TEOS to red-beet-pigment, generates a modified red-beet-pigment of the same color but less chromaticity.

The food models have the same tendency. The color difference between systems formed is: beverage-pigment and pigment: ∆E = 1.80; beverage-modified pigment and modified pigment: ∆E = 4.68 and finally beverage-pigment and beverage-modified pigment: ∆E = 4.13. In the yogurt model the color difference between yogurt-pigment and yogurt-modified pigment is ∆E = 5.91.

CIELab parameters of unmodified and modified red-beet-pigment and their application in beverage and yogurt model systems can be seen in Table 2. For powder samples it can be seen that there is a tendency to a red color in all samples with a small decrease in luminosity from 76.21 to 75.18 in this red from unmodified to modified pigments, the latter being slightly darker.

**Table 2:** Color analysis for unmodified and modified red beet pigments applied to beverage and yogurt model systems.

Sample	L *	a *	b *	C	Hº
Red-beet-pigment	76.21	36.86	1.54	36.90	2.39
Modified red-beet-pigment	75.18	39.10	2.45	39.17	3.58
Beverage–pigment	76.95	37.71	3.79	37.90	5.73
Beverage–modified pigment	74.87	34.43	2.36	34.51	3.92
Yogurt–red-beet-pigment	71.24	25.95	4.24	26.29	9.27
Yogurt–modified red-beet-pigment	68.58	21.03	2.3	21.15	6.24

L * = Luminosity; a * and b * are color values; C = chromaticity; and Hº = hue angle.

### 2.6. Infrared Spectroscopy Analysis

**Figure 6:**
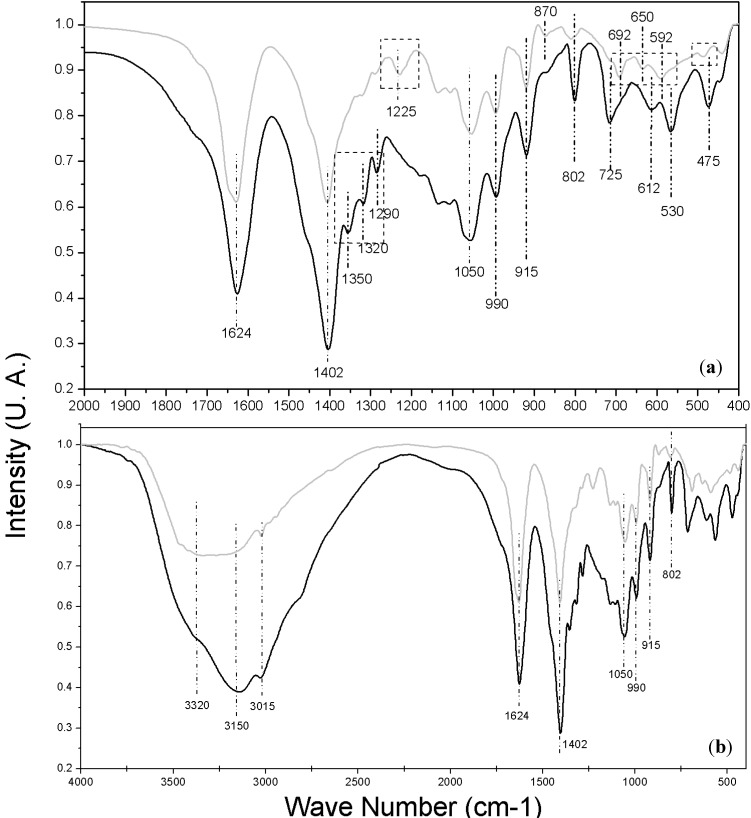
FT-IR Spectra Obtained; gray line for *Beta vulgaris* and black line for modified *Beta vulgaris*; (**a**) Zoom IR spectrum from 400 to 2000 cm^−1^ and (**b**) Full IR spectrum from 450 to 4000 cm^−1^.

A detailed chemical structure study of the modified pigment is beyond the scope of this paper. However, we performed a functional group analysis by Fourier Transform Infrared Spectroscopy (FTIR), as an approach to see how the incorporation of TEOS affects the original chemical structure of the pigment. Figure 6 shows the experimentally obtained infrared transmission bands of natural betalain (dark line) and modified betalain (gray line); Figure 6a is a magnification of the region from 400 to 2000 cm^−1^, there, the most interesting changes were observed–these changes are highlighted with rectangles in order to easily locate them. Some bands were unchanged; 1624, 1402, 1050, 990, 915, and 802 cm^−1^, therefore the functional groups or arrays of atoms and bonds responsible for these vibrational modes suffered no structural changes during the stabilization process. In contrast, other bands showed slight changes such as the 612 cm^−1^ band which was displaced to 650 cm^−1^ and the 475 cm^−1^ one to 480 cm^−1^. Additionally there were some significant changes such as those in the bands centered at 1350 cm^−1^, 1320 cm^−1^ and 1290 cm^−1^ appearing in the modified colorant spectra. The band centered at 1225 cm^−1^ in natural colorant spectra disappears after the stabilization process.

Assignments of bands with their corresponding vibrational modes were performed according to previous reports [39,40]. However it was not possible to relate all experimentally found bands with a vibrational mode using this method. Therefore computational calculations to achieve the correct and complete assignation of bands were necessary; these are shown in Table 3. The method and the basic set used were suitable for betalain molecules and yielded a good approximation of the experimental vibrational modes. These calculated band assignments were compared with the data reported in charts and tables to identify the type of vibration for each functional group [41], and to avoid errors in the vibrational band assignments.

Unchanged vibrational modes are associated with the benzene ring, pyridine functional group, and C-H groups, so we may conclude that the heterocyclic and benzene ring backbone are unchanged. However, there were changes in the neighboring atoms which modified the local vibrations of the 1350 cm^−1^ and 1290 cm^−1^ bands (Figure 6a) that were overlapped and hidden in the band at 1402 cm^−1^. These two bands correspond to C-H vibrational modes as shown in Table 4.

A similar case is the band centered at 1320 cm^−1^; it is attributed to the carboxyl group bonded to a heterocycle; here the neighboring oxygen atom was radically modified by exchanging its hydrogen for a -Si(OCH_3_)_3_ group, according to the reaction hypothesis. The strongest evidence of this hypothesis is provided by the appearance of the band centered at 530 cm^−1^ assigned to a vibrational mode of a C-O-Si group in agreement with DFT computational calculations. The modification of the close neighbors of the carboxyl also explains the disappearance of the bands centered at 1225, 692, 650, 592; and 480 cm^−1^ associated with the vibrational modes of the C-O; C=O; O=C-O; C=O; and C-COOH groups respectively.

**Table 3:** FTIR Assignment of the Most Important Bands Observed in the *Beta vulgaris* spectra.

Observed Infrared Bands (cm^−1^)	IR Bands Assignment According to [39,40] (ben-r, benzene ring)	Calculated Infrared Bands (cm^−1^)	IR Band Assignments According to B3LYP Calculations	Absorption Bandsin the Region According to Socrates [41]
3630	unreported	3620–3632	O-H stretching	O-H and N-H stretching
3150	unreported	3198	ben-r C-H asymmetric stretching	C-H stretching (unsaturated)
3015	unreported	3029	C-H symmetric stretching	C-H stretching (unsaturated)
1624	C=N stretching	1625	C-N-H of ben-r breathing motion	N-H deformation, primary amines. C-N stretching, (at 1650–1690 cm^−1^)
1402	C-H deformation	1414	C-H deformation	C-H symmetric deformation
1225	C-O stretching	1227	C-O stretching	C-O stretching, carboxylic acids
1050	C-O stretching	1052, 1080–1100	C-N-H of ben-r breathing motion (confirmation) C-O asymmetric stretching	C-O stretching
990	ben-r C-H deformation	991	C-H deformation from pyridine functional group	C-H in plane deformation
915	ben-r C-H deformation	921	ben-r C-H deformation	C-H in plane deformation at Pyridines
802	ben-r C-H deformation	798	ben-r C-H deformation	C-H deformation and rings deformation
692	unreported	698	C=O stretching C-O deformation	C-O out-of-plane deformation vib.
650	unreported	655	O=C-O scissoring bend	O-C=O in-plane deformation vib, usually at ~655 cm^−l^
592	unreported	592	C-O deformation C=O rocking	C=O deformation vib.
480 *	unreported	480	C-COOH rocking	CC=O deformation vib.
450 *	unreported	452	C-C=O rocking	C=O rocking vib.

* ben-r, benzene ring.

**Table 4:** Modification FTIR bands observed and tentative assignment for *Beta vulgaris* modified spectra.

Infrared Bands Modified (cm^−1^)	Observed Modification	Infrared Bands Calculated (cm^−1^)	IR Bands Assignment According to B3LYP Calculations
1350	show up	1352	C-H wagging bend
1320	show up	1312	C-COOH stretching
1290	show up	1288	C-H twisting bend
1225	fade out	1227	C-O stretching
725	show up	728	O-H rocking
692	fade out	698	C=O stretching C-O deformation
650	fade out	655	O=C-O scissoring bend and O-C=O in-plane deformation vib
612	show up	613	O-H rocking
592	fade out	698	C=O stretching C-O deformation
530	show up	538	C-O-Si stretching
480 *	fade out	480	C-COOH rocking
475	show up	470	Pyridine ring breathing motion

* ben-r; benzene ring.

### 2.7. Considerations on the Modified Pigment

This part of the discussion focuses on considerations about toxicity of the novel pigment. Silicon is present in foods as silicon dioxide (SiO_2_, silica) and silicates such as orthosilicic acid [Si(OH)_4_]. TEOS is the ethyl ester of orthosilicic acid, which is the most readily available source of silicon to man, also is the main chemical species by which silicon is absorbed after oral consumption—it is naturally present in drinking water and other liquids—[42]. Silicon, silicon dioxide and silicates are considered *quantum satis* or GRAS substances [43,44,45,46] so a daily required intake level is not well established.

However, the European Commission to the European Food Safety Authority, the Scientific Panel on Food Additives and Nutrient Sources added to Food has been asked to deliver a scientific opinion on calcium silicate, silicon dioxide and silicic acid gel added for nutritional purposes to food supplements, and they state that: a Safe Upper Level (UL) for daily consumption of silicon is 700 mg silicon/day for adults over a lifetime (equivalent to 12 mg silicon/kg body weight/day for a 60 kg adult). The EFSA Panel on Dietetic products, Nutrition and Allergies (NDA) was unable to set an UL for silicon, but estimated that the typical dietary intake of 20–50 mg silicon/day (equivalent to 0.3–0.8 mg/kg body weight/day in a 60 kg person) is unlikely to cause adverse effects [42]. Furthermore FDA recommends the maximum permitted amount of silicon dioxide as a direct additive to food is 2% by weight for ree-flow, anti-caking, microencapsulation, *etc.* [46].

## 3. Experimental Section

### 3.1. Chemicals and Materials

TEOS was supplied by Aldrich Chemical Co. (Mexico City, Mexico). Hydrochloric acid (HCl), ascorbic acid, ethylenediaminetetraacetic acid (EDTA), and sodium chloride were supplied by J.T Baker (Mexico City, Mexico). An Amberlite ion exchange resin was used to remove free sugar compounds using a packed column. Fresh red beets (*Beta vulgaris L. varrubra*), Chenopodiaceae family (Chenopodiaceae), Centrosperma species—collected from central Mexico—were used for pigment extraction.

### 3.2. Betalain Extraction

The red beets were selected, weighed without stems, washed and subsequently placed in boiling water (98 °C) for two minutes in order to inactivate periplasmic enzymes. Later, the beet was cut and its juice was quickly extracted by mechanical action using an Omega extractor (Omega, Harrisburg, PA, USA), volume measurement was obtained as well. The solids were removed from the beet juice through a mesh sieve filter and centrifugation (13,000 rpm) to start the modification process.

### 3.3. Chemical Modification and Free Sugar Removal

The chemical modification method is performed by two reactions; three round bottomed flasks with 100 mL each of previously filtered and centrifuged beet juice were mixed with 0.98, 1.96 and 2.94 mL of TEOS—as solution at 97% v/v—and stirred for an hour at room temperature. Then, the product of this first reaction was introduced to a packed column with the ionic resin (Amberlite IRA 958Cl) to remove free sugar compounds; flow rate was 32 mL/min (20BV/h) using acidified water at pH 4.5 as eluent. One hundred mL of the solution collected from the column for each of the TEOS treatment were once again mixed in their respective flask with 0.98, 1.96 and 2.94 mL of TEOS and stirred for an hour at room temperature. Subsequently, the product of the second reaction was filtered and dried, 5% w/w of EDTA were used as a chelation agent. Samples were labeled as BE_1_, BE_2_ and BE_3_ for 0.98 (0.97% v/v), 1.96 (1.92% v/v) and 2.94 (2.85% v/v) mL content of TEOS respectively. Chemical modification was confirmed by FT-IR spectra using a Bruker Vector 33 spectrometer (Bruker, Bucharest, Romania); the samples were analyzed in a range from 4000 to 450 cm^−1^.

### 3.4. Pigment Powder Samples

Ascorbic acid and aerosil (silicon oxide) were the ingredients used to prepare the dispersion to be spray-dried; 0.5% w/w of aerosil and 0.1% w/w of ascorbic acid were added to the second reaction product as dryer agents in order to obtain the final product. A magnetic stirring bar, rotating at 1000 rpm, was used for forty-five minutes before atomization to assure homogenization. The spray-drying process was performed in a laboratory scale Lab-plant Spray Dryer SD-Basic (North Yorkshire, UK) [47].

### 3.5. UV-Light, PH, Temperature, and Color Tests

Pigment powder (1 g) was initially diluted in potassium hydrogen phthalate buffer (100 mL). Using a DT-Mini-2 UV-Vis spectrophotometer (Ocean Optics, Inc., Dudenin, FL, USA) absorbance was obtained at 480 and 540 nm. Then, buffer was continuously added to obtain an absorbance within the range of 0.8 to 1 u.a. The pH was measured in these conditions and in all tests a consistently initial value of pH = 4 was obtained. Subsequently the sample was exposed to an extreme light-radiation, temperature and pH changes to measure its performance.

**Figure 7:**
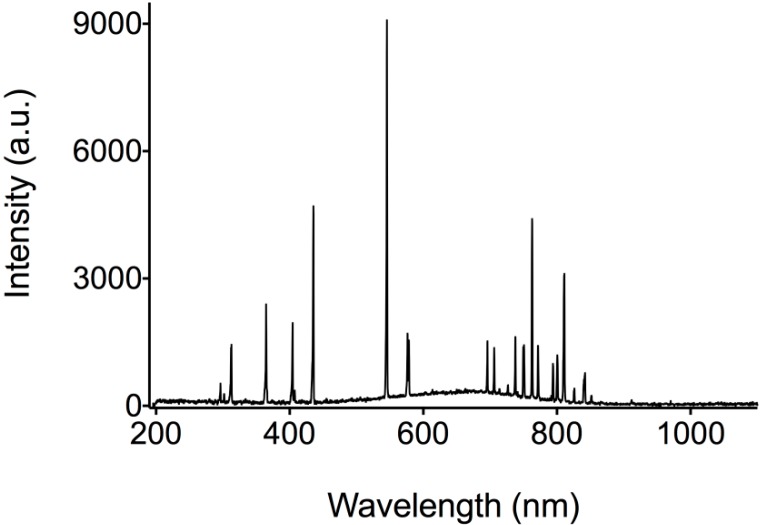
UV-Light source Spectrum.

UV-light performance test was made following ASTMD 4329 with some adjustments; the sample was illuminated continuously for 30 min with a UV-light source, whose spectrum can be seen in Figure 7. Absorbance was measured every 5 min to monitor the betalain concentration decay; distance between the sample and the UV source was 15 cm; the temperature was controlled at 25 °C; and the work area was kept isolated from other light sources.

For pH test, the betalain concentration at pH values of 4, 5, 6, and 7 was evaluated. In order to change the pH value, 0.1 M ammonium hydroxide (NH_4_OH) was added drop wise to every sample. Values of pH were monitored by using a UB-10 pH/mV meter (Denver Instrument, New York, NY, USA).

For the temperature tests the samples were exposed to thermal treatment at 60 °C using hot water and 0 °C using ice. Each sample was exposed for five minutes to heat and five minutes in cold to see betalain regeneration due to recondensation [48]. Five heat-cold cycles were performed.

For the UV-light, pH and temperature tests the absorbance measured at 480 and 540 nm never surpassed a value of 0.8 (+0.05). Betacyanin content was calculated as [49,50]:

(1) $${\text{B}}_{\text{c}}\left[{\text{mgL}}^{-1}\right]=\frac{\text{A}\times \text{F}\times \text{Mw}\times 100}{\text{ε}\times \text{l}}$$

where A is the absorption value at betanin λ_max (540 nm) corrected by the absorption at 600 nm, F is a dilution factor, Mw is the betanin molecular weight (500 g·mol^−1^), ε is the betanin molar extinction coefficient (60,000 L·mol^−1^·cm^−1^) and l is the path length (1.0 cm) of the cuvette.

### 3.6. Model Food Applications and Color Measurement

The model beverage was formulated using McIlvaine buffer solution containing 10% sucrose and selected amount of pigment (Sample BE_2_) (at pH 4.6). The McIlvaine buffers were made from 0.1 M citric acid and 0.2 M sodium phosphate dibasic in appropriate ratios for the required pH. Sucrose and pigment were dissolved in the buffer solution, mixed and poured into a 60 mL flask.

The model yogurt was prepared using 12 L of milk heated at 90 °C for five minutes, when the temperature was kept stable. Sucrose (1 kg) was added and separately another 5 L of milk were heated at 45 °C. The two heated milks were mixed and the temperature was maintained at 45 °C and finally 0.5 L of natural yogurt were added. The whole system was incubated during two hours to obtain the yogurt. A selected amount of pigment (Sample BE_2_) was added to the yogurt and poured into a 60 mL flask.

For the color test a model beverage and a model yogurt using unmodified and modified pigment power were prepared and the color measurement determined using a Miniscan EZ 4500 L colorimeter (HunterLab, Sunset Hill Road Reston, WV, USA) according to ASTM D1925. This colorimeter is based on two CIE color space version 1976 CIE (or CIELab). Calibration was performed on white color before sample analysis. The sample was placed filling up to the top an opaque plastic cylinder. Measurements were taken directly on the sample. Three shots were done for each measurement.

Color results were expressed as L, a *, b *, hue angle Hº = tan^−1^ (b *^2^/a *^2^) and chroma C = (a *^2^ + b *^2^)^1/2^. Hº indicates sample color (red = 0°/360°, yellow = 90°, green = 180° or blue = 270°) and C indicates color purity or saturation (color is more vivid as value increases). ∆E value is used as the total color difference between two samples and is calculated as [(∆L *)^2^ + (∆a *)^2^ + (∆b *)^2^]^1/2^.

### 3.7. Reducing Sugars, Phenol, and Antioxidant Content Tests

Antioxidant content using DPPH radical scavenging method, total phenolic content and total reducing sugar content were assessed to evaluate the nutritional content. For the measurement of the antioxidant content, the unmodified and modified pigment in solution were measured in terms of hydrogen donating or radical scavenging ability, using the stable radical DPPH. For each sample 100 µL are mixed with 2.90 mL of DPPH solution in 80% aqueous methanol; the mixtures were allowed to stand at room temperature in the dark for thirty minutes. The absorbance was read at 517 nm, and the total antioxidant content was calculated from a calibration curve using ascorbic acid as standard. Methanol was used to zero the spectrophotometer. The results are expressed as milligrams per liter of ascorbic acid equivalents (mg/L AAE).

The total phenolic content of each one of the unmodified and modified solution pigment were determined according to modified Folin-Ciocalteu method by Singleton and Rossi [51]. Each sample (0.2 mL) was mixed with Folin-Ciocalteu reagent (0.2 mL), then 7% aqueous Na_2_CO_3_ (2 mL) was added and mixed, and the final mixture was allowed to stand at room temperature in the dark for an hour. The absorbance was read at 750 nm, and the total phenolic concentration was calculated from a calibration curve using gallic acid as standard. The results are expressed as milligrams per gram of gallic acid equivalents (mg/g GAE).

The total reducing sugar content of each one of the solutions of unmodified and modified pigment were determined using Fehling’s A and B standard solutions. Fehling A and B solution are heated and stirred with a magnetic bar, an aqueous solution of 1.6% w/w of glucose is titrated in order to standardize the test as shown in Equation (2), where T is an titration factor, G are the milliliters of glucose solution employed and 0.016 is the concentration of glucose solution. Then, each sample was titrated and sugar-reducing contents were calculated as shown in Equation (3), where G' is the milliliters of sample used and F is a dilution factor if needed. The result GL is expressed as grams per hundred milliliters of sugar (g/100 mL glucose):

(2) T=0.016×G

(3) $${\text{G}}_{\text{L}}\left[\text{g}{\left(100\text{ mL}\right)}^{-1}\right]=\left(\frac{100\times \text{T}}{\text{G}\prime }\right)$$

### 3.8. Computational Details

Quantum chemical calculations for betalain molecules (Figure 2) were performed on a personal computer (PC) running the Gaussian 98W program [52]. The geometry was fully optimized assuming Cs point group symmetry using the Becke 3-Lee-Yang-Parr (B3LYP) and (LSDA) functional [53,54], supplemented with the standard 6-31 + G basis sets. Scaling of the force field was performed according to the Scaled Quantum Mechanical (SQM) procedure [53,54,55,56] using selective scaling in the natural internal coordinate representation [57].

**Figure 8:**
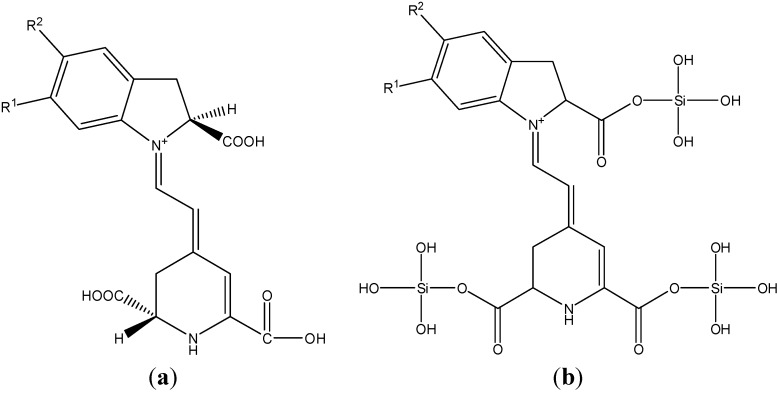
(**a**) Betalain molecule where R=OH was used for the chemical calculations approximation and (**b**) Modified simplified betalain molecule where R^2^ = R^1^ = OH was used for the chemical calculation approximations.

Once the simulation for the basic building blocks of the betalain molecule (Figure 8a) and another for a hypothetical modified molecule (Figure 8b) were obtained, the simulated IR spectra were plotted with the Gaussian View 5.0 package and the difference between the vibrational modes of the modified and unmodified betalain molecules were analyzed. Construction of the modified hypothetical molecule was obtained considering the reactivity of TEOS and the carboxyl group in an aqueous solution at pH less than 7.

## 4. Conclusions

We proposed to add tetraethyl orthosilicate (TEOS) to an acid solution of a pigment from *Beta vulgaris* to increase its stability. By this process, we obtained a novel modified pigment and studied the effects of TEOS on physicochemical properties as UV-light stability, pH stability and temperature stability. We performed and compared measurements of reducing sugars, phenol, and antioxidant contents on unmodified and modified red-beet-pigment, to prove its potential applications as an additive and pigment in the food industry. In relation to thermal attack, photo-light exhibition, and pH change the betalain degradation was 88.33%, 16.84% and 20.90% less, respectively, compared to the unmodified natural pigment. In all cases the sample BE_2_ showed a greater stability. Differences in vibrational modes that were found between unmodified and modified re-beet-pigments by FTIR, and the DFT calculations allow us to assume a possible chemical bond between TEOS and pigment molecules. This opens an opportunity for a detailed chemical analysis in further investigations. The assumption that this binding occurs by a sol-gel reaction mainly at the carbonyl groups creating a shield that protects the chromophore of molecule from UV-Vis photons, delaying its degradation and helping to protect it from temperature by a heat dissipation process is tempting and reasonable; that is why a thorough study of the chemical structural changes caused by TEOS on the red-beet-pigment is justified.

The tests for reducing sugars, antioxidant content, and phenol content performed on the sample BE_2_ showed reductions of 21.2%, 36.2% and 53.8%, respectively, suggesting the formation of a stable red-beet-pigment with a interesting content of reducing sugars, antioxidants and phenols which provides a higher nutritional value relative to a synthetic dye.

CIELab parameters of unmodified and modified red-beet-pigment showed that the color is preserved, however there is a small loss in the chromaticity parameter of the modified pigment. This is acceptable considering the improvement in stability and that therefore a long shelf life that can be achieved. In general the modified red-beet-pigment performance is good under extreme conditions thereby is promissory and an ideal pigment for drinks, yogurt and other uses in the food industry.
